# Inversion of Rice Biophysical Parameters Using Simulated Compact Polarimetric SAR C-Band Data

**DOI:** 10.3390/s18072271

**Published:** 2018-07-13

**Authors:** Xianyu Guo, Kun Li, Yun Shao, Zhiyong Wang, Hongyu Li, Zhi Yang, Long Liu, Shuli Wang

**Affiliations:** 1 College of Geomatics, Shandong University of Science and Technology, Qingdao 266590, China; guoxianyu1@126.com (X.G.); shuli.wang1@hotmail.com (S.W.); 2 Laboratory of Radar Remote Sensing Application Technology, Institute of Remote Sensing and Digital Earth (RADI), Chinese Academy of Sciences, Datun Road, Chaoyang District, Beijing 100101, China; shaoyun@radi.ac.cn (Y.S.); liulong@radi.ac.cn (L.L.); 3 Laboratory of Target Microwave Properties (LAMP), Zhongke Academy of Satellite Application in Deqing (DASA), Deqing 313200, China; 4 School of Earth Science and Resources, China University of Geosciences (Beijing), Beijing 100083, China; lhy1993@cugb.edu.cn; 5 Institute of Transmission and Transformation Engineering, China Electric Power Research Institute, Beijing 100055, China; yangzhi@radi.ac.cn

**Keywords:** compact-polarimetric SAR, rice biophysical parameters retrieval, *m-χ*/*m-δ* decomposition, modified water cloud model

## Abstract

Timely and accurate estimation of rice parameters plays a significant role in rice monitoring and yield forecasting for ensuring food security. Compact-polarimetric (CP) synthetic aperture radar (SAR), a good compromise between the dual- and quad-polarized SARs, is an important part of the new generation of Earth observation systems. In this paper, the ability of CP SAR data to retrieve rice biophysical parameters was explored using a modified water cloud model. The results showed that *S*_1_ was superior to other CP variables in rice height inversion with a coefficient of determination (*R^2^*) of 0.92 and a root-mean-square error (RMSE) of 5.81 cm. RL was the most suitable for inverting the volumetric water content of the rice canopy, with an *R^2^* of 0.95 and a RMSE of 0.31 kg/m^3^. The *m-χ* decomposition produced the highest accuracies for the ear biomass: *R^2^* was 0.89 and RMSE was 0.17 kg/m^2^. The highest accuracy of leaf area index (LAI) retrieval was obtained for RH (right circular transmit and horizontal linear receive) with an *R^2^* of 0.79 and a RMSE of 0.33. This study illustrated the capability of CP SAR data with respect to retrieval of rice biophysical parameters, especially for height, volumetric water content of the rice canopy, and ear biomass, and this mode may offer the best option for rice-monitoring applications because of swath coverage.

## 1. Introduction

Rice, one of the world’s leading food crops, occupies more than 11 percent of the world’s total arable land and provides food for more than half of the world’s population [1]. Rice biophysical parameters, such as the biomass, the crop height, the leaf area index (LAI), and the water content, are vital indicators of rice growth condition. For example, the dry biomass of rice ears is a direct manifestation of the rice yield. Rice crop height, an important index of rice phenology, is a comprehensive reflection of environmental factors, such as the climate, hydrology, and soil. The LAI is a key parameter in plant population and community growth analysis, and volumetric water content (VWC) can fully characterize any vegetation water shortage, both of which are closely related to vegetation growth. Therefore, timely and accurate estimation of rice biophysical properties is of great significance and consequently plays a role in forecasting rice productivity for ensuring regional or national food security. 

Synthetic aperture radar (SAR) has been proven to have a great potential for rice monitoring [2,3,4] and inversion of rice biophysical parameters [5,6] because of its all-weather day–night imaging capability, its ability to penetrate microwave signals, and the sensitivity of its signals to target properties. Erten et al. [5] dealt with the retrieval of agricultural rice height from space by using multi-polarization SAR images, and coherent and incoherent crop height estimation methods were discussed for the first time. Tan et al. [6] built a biomass inversion algorithm and inverted rice biomass, leaf area index, canopy height and related eco-physiological canopy variables, and the inversion results showed that the multitemporal SAR images at the C-band can be used to monitor the growth of rice. Although the application effect is very good, the swath coverage is small, and it is difficult to meet a wide range of application requirements. Compact-polarimetric (CP) SAR transmits on only one polarization and receives on two orthogonal polarizations, retaining their relative phase. The major motivation for compact polarimetry is to strive for quantitative applications of the same level as those from a fully polarized system, while avoiding the principal disadvantages (mass, power, and limited coverage) associated with a quad-polarized (quad-pol) SAR [7]. As a good compromise between the dual-polarized and quad-pol SARs, CP SAR is an important part of the new generation of Earth observation systems (EOS). RISAT-1, the first radar satellite with CP measurement capability, was successfully launched in April 2012 [8]. ALOS-2, launched in 2014, also includes CP as in the experimental imaging model in 2014 [9]. Moreover, in near future the RADARSAT Constellation Mission (RCM) of Canada will launch a C-band CP SAR satellite as well [10]. With the emergence of CP SARs of EOS, several studies have reported on the applications of CP SAR in rice monitoring such as rice mapping [11,12,13] and rice phenology [14,15,16].

However, the potential of CP SAR for the inversion of rice biophysical parameters has not been fully explored. Kumar et al. [17] found that the CP parameters are sensitive to the growth changes of wheat and corn, but did not look at the relationship between CP parameters and biophysical parameters. Zhang et al. [18] not only studied the response of rape to C-band CP SAR, they also carried out the inversion of rape biophysical parameters based on the random forest method, thus demonstrating the potential of CP SAR for the inversion of crop biophysical parameters. However, the physical mechanism of the crop was not taken into account by the inversion strategy. H. Yang et al. [19] devised a scattering index based on the *m-χ* decomposition components of simulated CP SAR data and constructed a regression model to obtain inversion results for oilseed rape biomass. The above studies indicated the capability of CP SAR in crop biophysical parameters inversion. However, the potential of CP SAR for the inversion of rice biophysical parameters has not been fully explored. Therefore, in this study, five temporal RADARSAT-2 fully polarimetric single look complex (SLC) C-band datasets were used to simulate CP SAR data, and 14 CP parameters, such as backscattering coefficients, Stokes vectors, and decomposition parameters, were extracted in each acquisition. Then, the capability of CP SAR data for the inversion of rice biophysical parameters was fully investigated by introducing a classic semi-empirical model, the water cloud model (WCM) [20], and a modified WCM (MWCM) in which the heterogeneity of the rice canopy and its phenological changes, as well as the double-bounce scattering between the rice canopy and the underlying surface were considered.

## 2. Study Site, Data Source, and Ground Measurement Campaign

**Figure 1:**
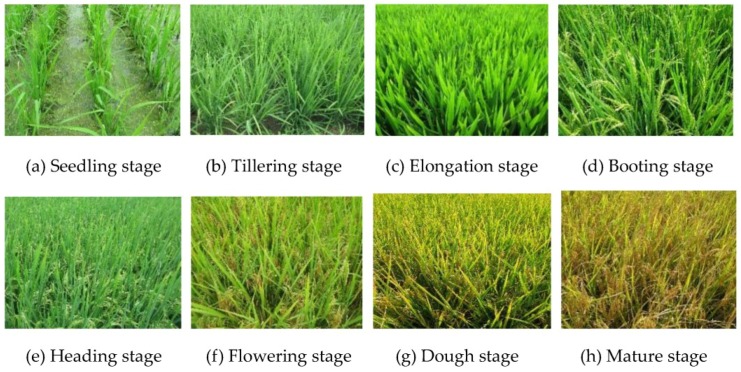
Photographs showing the eight phenological stages of indica rice in the field.

The study site was located at Jinhu, Xuyi, and Hongze, Jiangsu province (118°41′34′′~119°16′27′′ E, 33°17′05′′~33°56′39′′ N). Lacustrine plain is the main terrain type there, and so, the terrain is flat with an altitude of 5–9 m. The climate type is humid subtropical monsoon with abundant sunshine and rainfall. The site is one of the major grain-producing areas in the east of China, with rice being the main crop. The farmland plots in this area are large and produce one rice crop per year. The rice is at the growing stage from mid-June to late October or early November. The main types of rice in the region are hybrid and japonica, and the planting method used is transplanting and sowing. In this study, hybrid rice in a transplanted rice paddy was used. The phenological changes of indica rice are shown in Figure 1.

As the variations of biophysical parameters change with the development of the rice plants, five temporal RADARSAT-2 fully polarimetric single look complex (SLC) C-band datasets (Table 1), selected from 21 July to 15 October 2012, were used to simulate CP SAR data.

During the passage of the RADARSAT-2 satellite, field work was carried out. Thirty transplanted hybrid rice fields were selected as the sample plots for measuring the rice parameters. Each rice was recorded with an accurate global positioning system (GPS). Secondly, the rice parameters were measured from three rice plants, which were randomly selected in each rice field. The sizes of the rice plants (such as plant height, stem length and diameter, leaf length and width, ear length and width, etc.), the planting density, and other structural parameters involved in MWCM were measured with a steel ruler and a Vernier caliper. The LAI was measured using a SunScan Canopy analysis system [22]. The biomass and water content were obtained by destructive measurements of the sampled rice plants based on normal gravimetric processes. 

If volume scattering is the predominant mechanism responsible for the backscatter from vegetation, it seems appropriate to model a vegetation canopy as a cloud containing a volumetric water content (VWC) *m_v_* (kg/m^3^), which is defined as the water content per unit volume of the rice canopy [23]. Here, considering the heterogeneity of the rice canopy, the VWC of each layer in rice canopy, such as ear, leaf, stem, and so on, were proposed and calculated according to the definition of VWC:

(1) $$\begin{array}{c} m_{v\_e}=\;\left(F_{e}-D_{e}\right)N/h_{e}\\ m_{v\_s}=\;\left(F_{s}-D_{s}\right)N/h_{s}\\ m_{v\_l}=\;\left(F_{l}-D_{l}\right)N/h_{l}\\ m_{v}=\;\left(F-D\right)N/h \end{array}\}\;$$

where *N* (plants·m^−2^) is the density of the rice plants; *m_v_e_*, *m_v_s_*, *m_v_l_*, and *m_v_* are the VWC (ear, stem, leaf, and total, respectively); *F* (kg) is the fresh weight of the total plant, and *F_e_*, *F_s_*, and *F_l_* (kg) are the fresh weight of the ear, stem, and leaf of each rice plant, respectively; *D* (kg) is the dry weight of the total plant, and *D_e_*, *D_s_*, and *D_l_* (kg) are the dry weight of the ear, stem, and leaf of each rice plant, respectively; the difference between *F_i_* and *D_i_* (*i* = *e*, *s*, *l*) indicates the water content corresponding to each part of a rice plant, and *h_e_*, *h_s_*, *h_l_*, and *h* (m) are the height (ear, stem, leaf, and total).

## 3. Methodology

**Figure 2:**
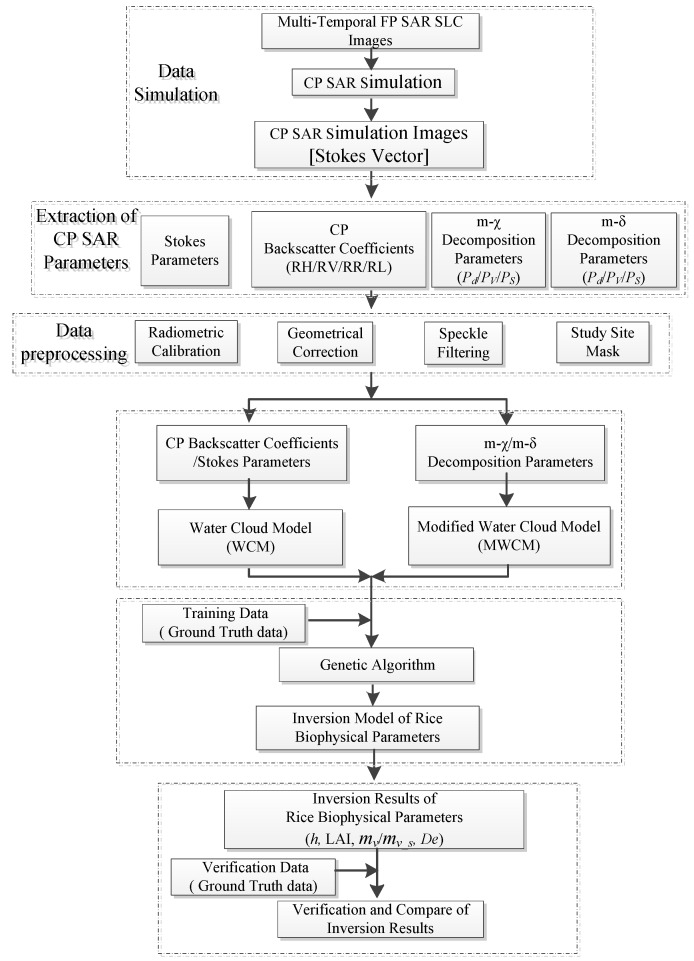
Methodology flow chart for the inversion of the rice biophysical parameters with the water cloud model (WCM) and modified WCM (MWCM) and compact-polarimetric (CP) synthetic aperture radar (SAR) parameters.

The flow chart of the methodology adopted in this study is shown in Figure 2. This methodology consisted mainly of the CP SAR data simulation, CP parameter extraction, data preprocessing, the inversion model building using the WCM/MWCM model, the genetic algorithm (GA), and finally, validation and analysis. 

### 3.1. CP SAR Data Simulation

The CP SAR data we used were simulated using the RADARSAT-2 full polarimetric (FP) SAR data as follows [24]: 

(1) The sensor emits right-hand circularly polarized microwave radiation, denoted *R*. Based on the Sinclair matrix for the FP SAR data [Γ], the electric vector, *E_B_*, of the target scattering field is constructed as follows:

(2) $$E_{B}=\left[\Gamma \right]R,R=\left(\frac{1}{\sqrt{2}}\right){\left[1,-j\right]}^{T},$$

(3) $$E_{B}=\left[\Gamma \right]R=\left(\frac{1}{\sqrt{2}}\right){\left[S_{HH}-jS_{HV},S_{HV}-jS_{VV}\right]}^{T},$$

where *S_HH_*, *S_HV_*, and *S_VV_* are the elements of the Sinclair matrix for the FP SAR data.

(2) The *E_H_* and *E_V_* components are calculated in the case where the antenna sends and receives linear polarization (*H* and *V*) signal as follows:

(4) $$E_{H}=\left[\begin{array}{cc} 1 & 0 \end{array}\right]E_{B}=\left(\frac{1}{\sqrt{2}}\right)\left(S_{HH}-jS_{HV}\right),$$

(5) $$E_{V}=\left[\begin{array}{cc} 0 & 1 \end{array}\right]E_{B}=\left(\frac{1}{\sqrt{2}}\right)\left(S_{HV}-jS_{VV}\right).$$

(3) The four elements of the coherence matrix *J* are calculated as follows:

(6) $$2J_{HH}=\langle {\left|S_{HH}\right|}^{2}\rangle +\langle {\left|S_{HV}\right|}^{2}\rangle +j\langle S_{HH}S_{HV}^{*}\rangle -j\langle S_{HV}S_{HH}^{*}\rangle ,$$

(7) $$2J_{HV}=\langle S_{HH}S_{HV}^{*}\rangle -\langle S_{HV}S_{VV}^{*}\rangle -j\langle {\left|S_{HV}\right|}^{2}\rangle -j\langle S_{HH}S_{VV}^{*}\rangle ,$$

(8) $$J_{VH}=J_{HV}^{*},$$

(9) $$2J_{VV}=\langle {\left|S_{VV}\right|}^{2}\rangle +\langle {\left|S_{HV}\right|}^{2}\rangle -j\langle S_{VV}S_{HV}^{*}\rangle +j\langle S_{HV}S_{VV}^{*}\rangle .$$

(4) Based on the elements of matrix *J*, the Stokes vector, *S*, is calculated as follows:

(10) $$S_{1}=J_{HH}+J_{VV},$$

(11) $$S_{2}=J_{HH}-J_{VV},$$

(12) S3=Re{〈SHHSHV∗〉+〈SHVSVV∗〉}−Im〈SHHSVV∗〉,

(13) S4=−Im{〈SHHSHV∗〉−〈SHVSVV∗〉}−Re〈SHHSVV∗〉+〈|SHV|2〉,

where *S*_1_, *S*_2_, *S*_3_, and *S*_4_ are the four elements of the Stokes vector.

(5) According to the relation between the Sinclair matrix [Γ] and the covariance matrix, *C*_3_, a relation between the matrix elements of the FP data and the Stokes vector for the CP mode is established as follows:

(14) S1=1/2C11+1/2C22+1/2C33+(1/2)ImC12+(1/2)ImC23,

(15) S2=1/2C11−1/2C33+(1/2)ImC12−(1/2)ImC23,

(16) S3=(1/2)ReC12+(1/2)ReC23−ImC13,

(17) S4=−ReC13−1/2C22−(1/2)ImC12+(1/2)ReC23.

**Figure 3:**
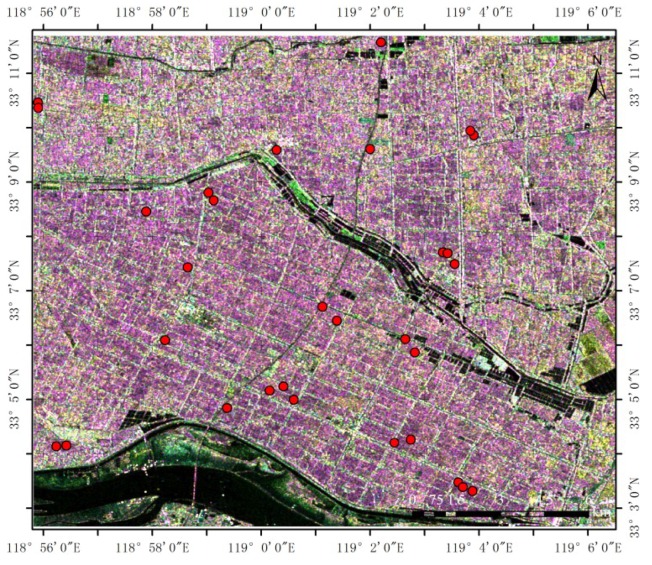
The simulated CP SAR data of 21 July 2012—color composite images of CP backscattering coefficients (R = RR (right circular transmit and right circular receive), G = RV (right circular transmit and horizontal linear receive), and B = RH (right circular transmit and horizontal linear receive)). The red circles in the figure represents 30 typical rice fields as the experimental area.

The simulated CP SAR data were transmitted as right-circular polarization (R) and received as horizontal (H) and vertical (V) polarizations, with a resolution of 30 m and a noise floor of −25 dB. Figure 3 shows the color composite images of the CP backscattering coefficients generated from the simulated CP SAR data.

### 3.2. Extraction of CP SAR Parameters and Data Preprocessing

Following the process described in Section 3.1, the simulated CP SAR data was stored in Stokes vector *S* = {*S*_1_*, S*_2_*, S*_3_*, S*_4_}. A total of 14 CP parameters, including Stokes vectors, four backscattering coefficients, three *m-δ* decomposition parameters, and three m–χ decomposition parameters, were extracted in each acquisition, as described in Section 2. A 5 × 5 frost filter was applied to all the CP parameters, and the resulting images were georectified. Then, image subsets were taken within the boundaries of the study site, and the areas designated as urban, water, road, forest, and other land cover types were removed. Only the areas classified as hybrid rice were extracted for the inversion of the rice biophysical parameters.

**Figure 4:**
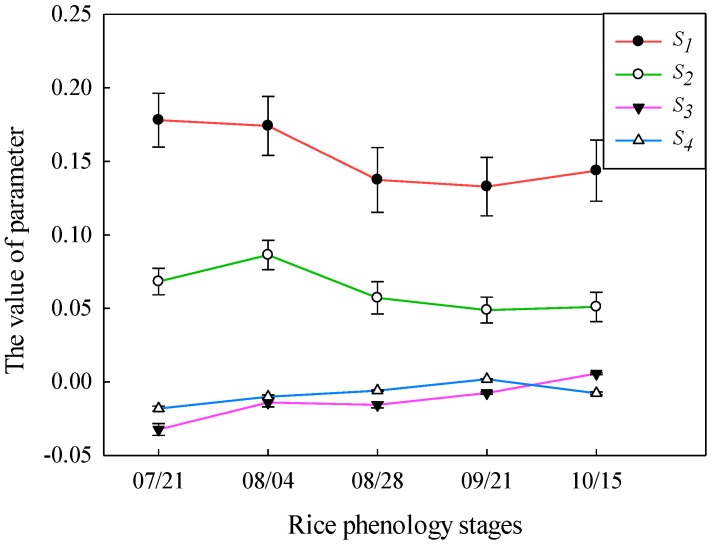
The temporal behaviors of the four Stokes parameters from the elongation stage to the mature stage.

The four Stokes parameters represent the intensity and polarization state of the radar echoes. *S*_1_ and *S*_2_ are closely related to the overall backscatter. Figure 4 shows the temporal behaviors of the four Stokes parameters from the elongation stage to the mature stage. It can be seen that the energy is mainly concentrated in the *S*_1_ and *S*_2_ components, whereas the contributions of *S*_3_ and *S*_4_ are very small. Although there were significant changes in the rice biophysical parameters during rice growth, the energy values of *S*_3_ and *S*_4_ were close to zero and did not change much during each rice phenology stage. It can be seen that *S*_3_ and *S*_4_ are not sensitive to the changes in rice growth and biophysical parameters. Therefore, *S*_3_ and *S*_4_ are not suitable for the inversion of the rice biophysical parameters. For *S*_1_ and *S*_2_, from the elongation stage to the booting stage, the rice height increased, and the water content of the rice also increased. In addition, the leaves widened and enlarged. Moreover, the values of *S*_1_ and *S*_2_ also closely reached the maximum values. However, from the booting stage to the heading stage, the dough stage, and the mature stage, the appearance of the rice ear and the change in leaf color and the underlay surface of the rice resulted in the decrease of the value of *S*_1_ and *S*_2_. Therefore, *S*_1_ and *S*_2_ deserve to be used to invert the rice biophysical parameters.

CP backscattering parameters have been proven to have a great potential in crop parameters’ inversion [18,19]. Four CP backscattering coefficients were simulated respectively in the RH (right circular transmit and horizontal linear receive), RV (right circular transmit and horizontal linear receive), RR (right circular transmit and right circular receive), and RL (right circular transmit and left circular receive) from the fully-polarized Stokes vector *S*. The calculation details are described by Equations (18)–(21) as follows:

(18) $$\sigma_{\mathrm{RH}}^{0}=\left[1,1,0,0\right]\times S,$$

(19) $$\sigma_{\mathrm{RV}}^{0}=\left[1,-1,0,0\right]\times S,$$

(20) $$\sigma_{\mathrm{RR}}^{0}=\left[1,0,0,-1\right]\times S,$$

(21) $$\sigma_{\mathrm{RL}}^{0}=\left[1,0,0,1\right]\times S.$$

**Figure 5:**
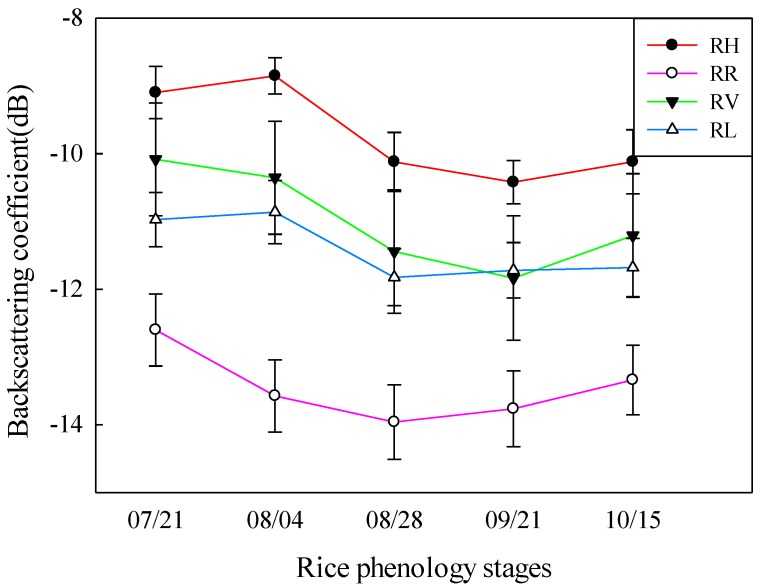
The temporal behaviors of the CP backscattering coefficients in the RH, RV, RR and RL polarizations from elongation stage to mature stage.

Figure 5 shows the temporal behaviors of the CP backscattering coefficients in the RH, RV, RR, and RL polarizations from the elongation stage to the mature stage. All the backscatter powers were high values at the elongation stage. The backscattering coefficients of RV dropped sharply in the booting stage, and the other three backscatter values showed little difference. The increase in rice height resulted in high attenuation of vertically-polarized return. From the booting stage to the heading, dough, and mature stages, the backscattering coefficients of RV had declined slowly. This was because the rice had grown to a certain height so that degree of attenuation of vertically-polarized return had not changed much. From the booting stage to the heading and dough stages, the backscattering coefficients of RH\RR\RV declined dramatically, and the increase in the rice leave layer and the appearance of the rice ear layer resulted in high attenuation of horizontal-polarized return. Moreover, as the leaves withered and turned yellow gradually, the volume scattering and double-bounce scattering were decreased, and the backscattering power of RH\RR\RV decreased gradually as well. With rice leaves continuing to fall off and wither, although the volume scattering decreased, the double-bounce scattering surface scattering from the underlying surface increased. Therefore, from the dough to the mature stage, all the backscatter powers increased slowly with a gentle slope. All the backscattering coefficients were excellent indicators for different phenological stages of rice and also had great potential for the inversion of the rice biophysical parameters.

The three scattering components of the *m-χ* and *m-δ* decomposition, the physical meanings of which were similar to that of the Freeman–Durden decomposition for full polarimetric SAR, were generated from the simulated CP SAR data as described by Equations (22)–(26) [25] as follows:

(22) $$m=\sqrt{\left(S_{2}^{2}+S_{3}^{2}+S_{4}^{2}\right)}/S_{1},$$

(23) $$\sin2\chi =-S_{4}/\left(mS_{1}\right),$$

(24) $$\delta =\arg(S_{3}+jS_{4}),$$

where *m* is the degree of polarization, *δ* is the relative phase, and *χ* is the ellipticity angle of the Poincaré sphere. They are all the child parameters of the Stokes vector. The degree of polarization *m* is negatively correlated with the scattering entropy; the greater the scattering entropy, the smaller the *m*. The phase angle *δ*, which is the relative phase angle between the RH and RV polarizations in CP SAR data, includes the phase information of the backscatter of the targets. The sign of the ellipticity angle *χ* is an unambiguous indicator of even versus odd bounce backscatter, and sin2*χ* is known formally as the degree of circularity [26]. 

The Stokes parameter *S*_1_, which is the total backscattered energy received by the CP systems, in combination with the CP parameters *m*, *δ*, and *χ* were used to generate the *m-δ* (as shown in Equation (25)) and *m-χ* decompositions (as shown in Equation (26)), respectively. *P_d_*, *P_v_*, and *P_s_* correspond to the double-bounce, the randomly polarized constituent (volume), and single-bounce (and Bragg) backscattering.

(25) $${\left[\begin{array}{c} P_{d}\\ P_{v}\\ p_{s} \end{array}\right]}_{m-\delta }=\left[\begin{array}{c} \sqrt{mS_{1}\left(1+\sin\delta \right)/2}\\ \sqrt{S_{1}\left(1-m\right)}\\ \sqrt{mS_{1}\left(1-\sin\delta \right)/2} \end{array}\right],$$

(26) $${\left[\begin{array}{c} P_{d}\\ P_{v}\\ p_{s} \end{array}\right]}_{m-\chi }=\left[\begin{array}{c} \sqrt{mS_{1}\left(1-\sin2\chi \right)/2}\\ \sqrt{S_{1}\left(1-m\right)}\\ \sqrt{mS_{1}\left(1+\sin2\chi \right)/2} \end{array}\right].$$

**Figure 6:**
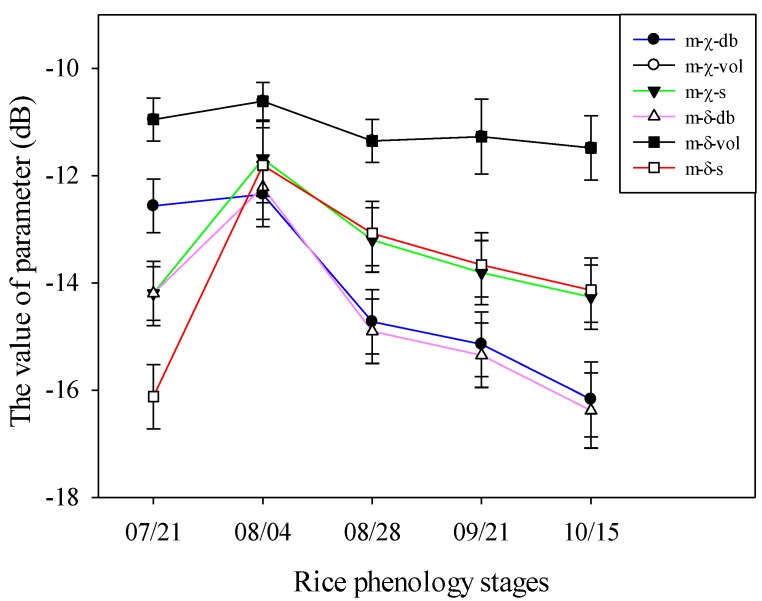
The temporal behaviors of the three scattering components of the *m-χ* and *m-δ* decomposition from the elongation stage to the mature stage, db = double bounce, vol = volume, and s = surface.

Figure 6 shows the temporal behaviors of the three scattering components of the *m-χ* and *m-δ* decomposition from the elongation stage to the mature stage. It can be seen that the main scattering mechanism for the reflected radar signal is multiple scattering (volume scattering), which is much higher than the even scattering (double-bounce scattering) and single scattering (surface scattering). As the rice crop was in the elongation stage on 21 July, it was short, and the underlay surface was mostly water, which prompted the three kinds of scattering to be weak. With the growth of the rice, the rice biophysical parameters, such as rice height, biomass, and water content, increased on 4 August (booting stage), which resulted in the increase of the three kinds of scattering. However, from heading stage to the dough and the mature stages, with the emergence, growth, and maturity of the rice ear, the leaves turned yellow gradually, the rice water content decreased, and the rice biomass increased, which made the three kinds of scattering energy decline. It can be seen that with the growth of the rice and the change of the rice biophysical parameters, the three scattering energies of the rice also changed to varying degrees. Therefore, the scattering parameters are very sensitive to the rice biophysical parameters in the rice growth, which provides a possibility for building a model relationship between the rice biophysical parameters and the CP decomposition parameters.

### 3.3. The Inversion Model of Rice Parameters Building with CP SAR

The CP parameters, including the backscattering coefficients, the Stokes parameters, and the three scattering components of the *m-χ* and *m-δ* decomposition, was investigated for the inversion of the rice biophysical parameters by introducing a classic semi-empirical model, the water cloud model (WCM) [20,23], and a modified WCM (MWCM) in which the heterogeneity of the rice canopy and its phenological changes, as well as the double-bounce scattering between the rice canopy and the underlying surface, were considered [27].

#### 3.3.1. The Inversion Model of Rice Parameters Using CP Backscatter and WCM

The WCM is one of the most popular semi-empirical models. It was first proposed by Attema [23] and Ulaby et al. [20] and was extensively applied to the inversion problems of radar remote sensing [28,29,30]. It treats the canopy as a water cloud, consisting of a collection of identical water particles, characterized by a uniform scattering phase function [23]. Ignoring second-order contributions resulting from multiple scattering between the canopy particles and the soil surface, the backscattering coefficient of the canopy is given by the following equation:

(27) $${\sigma_{can}}^{0}(\theta )={\sigma_{veg}}^{0}(\theta )+\sigma_{s}^{0}(\theta ),$$

where ${\sigma_{veg}}^{0}(\theta )$ is the contribution of the vegetation volume, $\sigma_{s}^{0}\left(\theta \right)$ is the backscattering contribution of the soil surface in the presence of vegetation cover, and θ is the angle of incidence relative to nadir [20].

With respect to the rice canopy, as there is a large difference of volumetric water content between the leaf and stem layer in the rice canopy, it was assumed to consist of two layers: an upper layer of height *h*_1_, dominated by leaves, and a lower layer of height *h*_2_, dominated by stem. We established the scattering energy from each layer of the rice canopy in the framework of the WCM to express the rice biophysical parameters:

(28) $${\sigma_{can}}^{0}(\theta )=\sigma_{l}^{0}(\theta )+{\sigma_{st}}^{0}(\theta )+\sigma_{s}^{0}(\theta ),$$

(29) $$\sigma_{l}^{0}(\theta )=A_{l}\cdot (1-\exp(-B_{l}L/h_{1}))\cos(\theta )\cdot (1-\gamma_{l}^{2}(\theta )),$$

(30) $${\sigma_{st}}^{0}(\theta )=A_{st}(\theta )m_{v}\cdot h_{2}\cdot \gamma_{l}^{2}(\theta ),$$

(31) $$\sigma_{s}^{0}(\theta )=C_{S}(\theta )m_{s}\cdot \gamma_{st}^{2}(\theta )\gamma_{l}^{2}(\theta ),$$

(32) $$\gamma_{l}^{2}(\theta )=\exp(-2\alpha_{l}L\sec(\theta )),$$

(33) $$\gamma_{st}^{2}(\theta )=\exp(-\alpha_{st}m_{v}h_{2}),$$

where *θ* is the incidence angle of the CP SAR data. The parameters with subscript *l* are the parameters related to the leaves layer of the rice, and the parameters with subscript *st* are the parameters related to the stem layer of the rice. Moreover, the parameters with subscript *s* are the parameters related to the soil layer of rice. Because *h*_1_ and *h*_2_ are proportional to the total plant height *h*, and *h*_1_ and *h*_2_ were replaced with *h* in Equations (29), (30), and (33). *L* is the LAI, and *γ* is the transmission coefficient. However, the WCM treats the canopy as a water cloud, consisting of a collection of identical water particles, characterized by a uniform scattering phase function. So, the VWC of the rice canopy (*m_v_*) is identical to that of the stem layer (*m_v_s_*). It can be seen that Equation (28) contains six model coefficients (*A_st_*, *A_l_*, *B_l_ C_s_*, *α_st_*, and *α_l_*) and four rice biophysical parameters (*L*, *h*, *m_s_* and *m_v_*).

As the CP backscattering coefficients and Stokes parameters are the power of the backscatter wave, they can replace $\sigma_{can}^{0}$ and be expressed as a function of the rice biophysical parameters by substituting Equations (28)–(33) into Equation (27). For each equation for $\sigma_{can}^{0}\left(\theta \right)$, there is an ill-posed problem. Therefore, it is obviously impossible to solve for four rice biophysical parameters in one equation. Therefore, to solve for one of the parameters, the values of the other three parameters must be assumed to ensure that one equation can be used to calculate the value of a rice biophysical parameter. Taking the seedling stage to the heading stage as an example, we take *h* as the parameter to be solved, and the other three parameters must be assumed. From the seedling stage to the heading stage, the underlying surface is calm water, so *m_s_* is assumed to be 1. The quantity *m_v_h*_1_ for the top layer is related to the wet and dry biomasses of the leaves, which in turn are related to the green LAI [20]. So, we can substitute *m_v_h*_1_ for the LAI in Equations (29) and (32). In each phenological stage, we measured the *m_v_* of the sample plots, and the entire image was interpolated by the nearest neighbor interpolation algorithm using the measured *m_v_* so that each pixel had a value of *m_v_*. So, when every pixel in the image was used Equation (28) to calculate *h*, the *m_v_* was replaced by corresponding value. Therefore, an unknown parameter (*h*) has only one equation to solve for *h.* When solving for other parameters, such as the LAI, we assume that the other three rice parameters are specific values according to the same method so as to effectively solve for the LAI. 

#### 3.3.2. Construction of the MWCM

In order to further investigate the capability of CP SAR data in the rice parameters inversion, especially the CP decomposition parameters, such as surface, volume, and double-bounce scattering, a modified WCM (MWCM) was introduced in which the heterogeneity of the rice canopy and its phenological changes, as well as the double-bounce scattering between the rice canopy and the underlying surface were considered [27]. In the MWCM, the rice canopy was assumed to consist of many scattering cells in the horizontal direction, containing the rice and space part. A volume fraction coefficient *F* and a water content fraction coefficient *n* are defined to quantitatively describe the heterogeneity of the rice canopy in the horizontal direction. While in the vertical direction, the rice canopy was divided into leaves, stems, and ears. The multi-layers, parts, and their volumetric water content changes with the phenological stages.

In the frame work of the MWCM, ten kinds of scattering mechanisms of the rice canopy were considered during the whole growing season, in which four periods were included (Table 2). In the first period, the seedling stage, because the rice stem is short and the leaves are small, the rice part can be regarded as a whole. However, no leaves were distributed in the space part, and the water content in the space part is negligible. Therefore, there are four kinds of scattering, including the underlying surface scattering through the rice part (*S_g_r_*), the underlying surface scattering through the space part (*S_g_s_*), the volume scattering from the rice part (*V_f_r_*), and the double-bounce scattering between the underlying surface and the rice part (*D_g_f_*). From the tillering stage to booting stage, the leaves and the stems grew longer, the water content of the space part increased, and the water contents of the stem layer and the leaf layer were different. So, there are three additional scattering mechanisms, including the volume scattering from the leaf layer in the space part (*V_f_s_*), the surface scattering from the stem layer (*S_t_*), and the double-bounce scattering between the underlying surface and the stem (*D_g_t_*). From the heading stage to the flowering stage, due to the growth of the rice ear, the rice growth reached its peak and the underlying surface of the rice was covered by the plant, and so, the double-bounce scattering between the leaves layer and the underlying surface is negligible. Moreover, the volume scattering from the ear layer in the rice part (*V_e_r_*) and the double-bounce scattering between the underlying surface and the ear layer (*D_e_s_*) is considered. From the dough stage to the mature stage, some of the rice ear moves to the space part due to increase of the biomass of the rice ear. Therefore, it is then necessary to increase the volume scattering (*V_e_s_*) of the space part. Table 2 shows the rice scattering mechanisms for different the phenological stages. All the 10 scattering mechanisms can be expressed as a function of the rice biophysical parameters with 16 model coefficients, including *F*, *n*_1_, *n*_2_, *A_e_*_1_*(θ)*, A*_e_*_2_*(θ)*, *A_f_*_1_, *B_f_*_1_, *A_f_*_2_, *B_f_*_2_, *A_t_*_1_, *A_t_*_2_, *C_g_*_1_*(θ)*, *C_g_*_2_*(θ)*, *α_f_*, *α_t_*, and *α_e_*, the physical meanings of which were described in [27].

#### 3.3.3. Coupling the CP Parameters from the *m-χ* and *m-δ* Decomposition with the MWCM.

With the CP decomposition, the surface scattering *P_s_*, the double-bounce scattering *P_d_*, and the volume scattering *P_v_* are calculated. Based on the MWCM, three equations relating the three scattering components to the rice variables could be built as follows:

(34) $${\left[\begin{array}{c} P_{d}\\ p_{v}\\ P_{s} \end{array}\right]}_{m-\chi }=\left[\begin{array}{c} D_{g\_e}+D_{g\_t}+D_{g\_f}\\ V_{e\_r}+V_{e\_s}+V_{f\_r}+V_{f\_s}\\ S_{g\_r}+S_{g\_s}+S_{t} \end{array}\right],$$

(35) $${\left[\begin{array}{c} P_{d}\\ p_{v}\\ P_{s} \end{array}\right]}_{m-\delta }=\left[\begin{array}{c} D_{g\_e}+D_{g\_t}+D_{g\_f}\\ V_{e\_r}+V_{e\_s}+V_{f\_r}+V_{f\_s}\\ S_{g\_r}+S_{g\_s}+S_{t} \end{array}\right],$$

where the parameters of *V*, *D*, and *S* with subscripts can be expressed as a function of the rice parameters (i.e., *D_e_*, LAI, *h*, *m_v_s_*, and *m_s_*) by introducing 16 model coefficients [27].

However, only three rice variables could be estimated simultaneously from Equation (34) or (35), which leads to the ill-posed problem of inversion. Therefore, as for the MWCM, the different phenological stages were assumed. Before the heading stage, no rice grains have been formed, and so, the rice biomass *D_e_* is 0. The underlying surface throughout these stages is water, and so, the moisture content of the underlying surface, *m_s_*, is 1. Therefore, there are only three rice parameters, that LAI, *h*, and *m_v_s_*, which could be calculated easily. From the heading to the mature stage, the rice particles have been presented, and the underlying surface of the rice field is wet soil. Two hypotheses were applied as follows: (1) assume that the backscattering from the underlying surface is constant so that *m_s_* can be eliminated; and (2) the stem layer is ignored considering that the attenuation from the ear and the leaf layers are large after the heading stage, which results in the variable *m_v_* being eliminated. Three unknown rice parameters (the LAI, *h*, and *D_e_*) can thus be calculated using Equations (34) or (35). 

#### 3.3.4. Calculating the Model Parameters Based on the GA of Rice Characteristics

A genetic algorithm [31] was used to solve Equations (28), (33), and (34) for estimating the rice biophysical parameters in five phenological stages. A total of 10 rice fields, distributed uniformly in the study area, were used as training data for the model training, and the remaining twenty rice fields were used for validation. The specific steps were as follows.

(1) Initialization and Genetic Representation

As mentioned in Section 3.3.1 and Section 3.3.2, there are six and 16 model coefficients in the inversion models of the WCM (Equation (28)) and the MWCM (Equations (33) or (34)), respectively. In the genetic representation of the GA, each coefficient of Equations (28) and (33)/(34) was encoded by using binary coding as the gene substring.

In the WCM, the initial values and ranges of four model coefficients were initialized by solving the WCM with a set of training data. For example, the attenuation coefficients of the leaf layer and the stem layer, *α_l_* and *α_st_*, respectively, were initialized to [0, 4] and [0, 2], respectively.

As to the MWCM, the initial values and ranges of the volume fraction coefficient *F* and VWC fraction coefficient *n_i_* were determined by the ground data measurements and empirical assumption. In terms of the other coefficients, their initial values and ranges were initialized by solving the MWCM with a set of training data. For example, the attenuation coefficients *α_f_* and *α_e_* were initialized to [0, 1] and [0, 4], respectively. 

Based on their ranges, the gene substring lengths of the coefficients were determined when the desired solution accuracy to four decimal places was considered. Then, the length of the chromosome (total of the gene substring) was determined (i.e., for the inversion model of the WCM, 66 from the elongation (21 July) to the booting stage (4 August), 51 in the heading stage (28 August), and 55 from the dough (21 September) to the mature stages (15 October); In the inversion model using the MWCM, 175 from the elongation (21 July) to the booting stage (4 August), 135 in the heading stage (28 August), and 149 from the dough (21 September) to the mature stages (15 October)). The population of the coefficients was generated using a random generator, and the population size of 100 was initialized.

(2) Reproduction

The chromosomes generated in the initial population were then chosen for participation in the reproduction process based on their fitness values [32]. In this study, the fitness value was calculated as Equations (36)–(38) and proportionate fitness selection was used. A chromosome with a higher fitness value had a higher probability of being copied into the cross-over pool.

(36) $$f=\frac{1}{1+SSE},$$

(37) $$For\;WCM,\;SSE=\sum_{i=1}^{m}\left[{\left(S_{i,est}-S_{i,obs}\right)}^{2}\right]\;or\;\sum_{i=1}^{m}\left[{\left(\sigma_{i,est}^{0}-\sigma_{i,obs}^{0}\right)}^{2}\right],\;$$

(38) $$For\;MWCM,SSE={\sum_{i=1}^{m}\left[{\left(P_{v,obs,i}-P_{v,est,i}\right)}^{2}+{\left(P_{d,obs,i}-P_{d,est,i}\right)}^{2}+{\left(P_{s,obs,i}-P_{s,est,i}\right)}^{2}\right]}_{m-\chi \;or\;m-\delta },$$

where $\sigma_{\;i,est\;}^{0}$ is the estimated backscattering coefficient for the *i*th training sample, $\sigma_{\;i,obs\;}^{0}$ is the backscattering coefficient for the observed value of the *i*th training sample, $S_{i,est}$ is the estimated Stokes scattering parameters for the *i*th training sample, and $S_{i,obs}$ is the observed value of the Stokes parameters of the *i*th training sample. $P_{v,est,i}$, $P_{d,est,i}$, and $P_{s,est,i}$ represent the decomposition of the three scattering components for the *i*th training sample estimates of the scattering component values; $P_{v,obs,i}$, $P_{d,obs,i}$ and $P_{s,obs,i}$ represent the decomposition of the three scattering components to observe the scattering of the *i*th training sample component values. *m* is the number of training samples*.*

(3) Cross-over

Cross-over is a recombinant operator that selects two chromosomes from the cross-over pool at random and cuts them into bits at a randomly chosen position [31]. The number of strings participating in mating depended on the cross-over probability. In this study, the cross-over probability was assumed to 0.9, and one-point cross-over was selected.

(4) Mutation

Mutation is an important process that permits new genetic material to be introduced to a population. A mutation probability is specified that permits random mutations to be made to individual genes (e.g., changing 1 to 0, and vice versa, for binary GAs). The mutation probability of 0.01 was selected in this study.

(5) Termination Criteria

Finally, the termination criterion of the GA process was determined. The GA process could be stopped when the fitness criterion was satisfied or the maximum number of generations was exceeded. In this study, the maximum number of generations was 2000.

## 4. Results and Discussions

**Figure 7:**
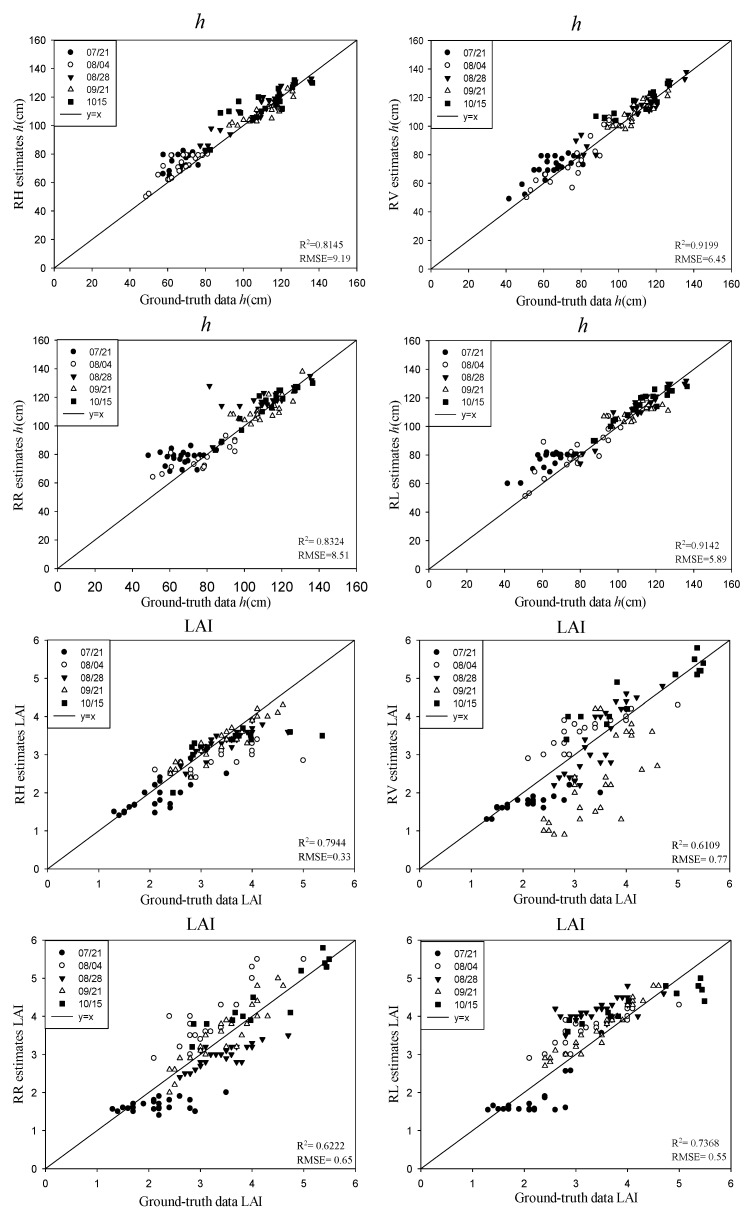

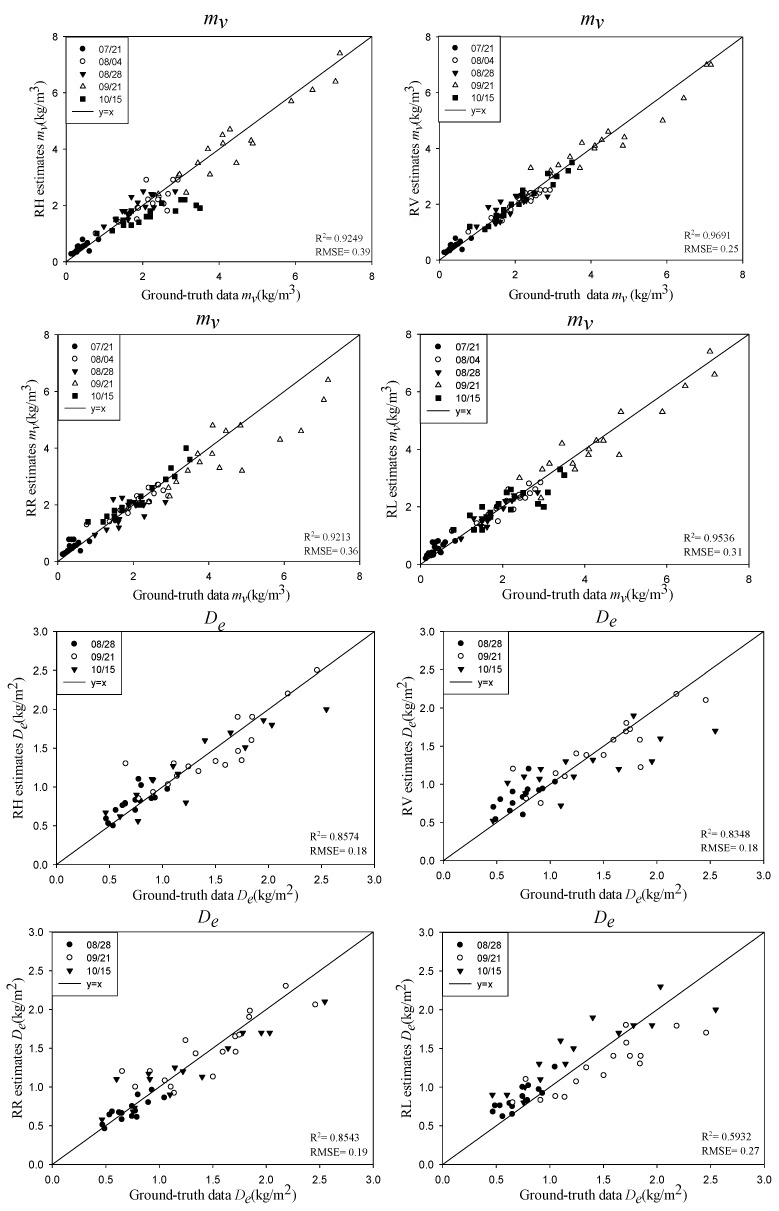
Results for the rice biophysical parameters using the CP backscattering coefficients for the different phenological stages of rice.

**Figure 8:**
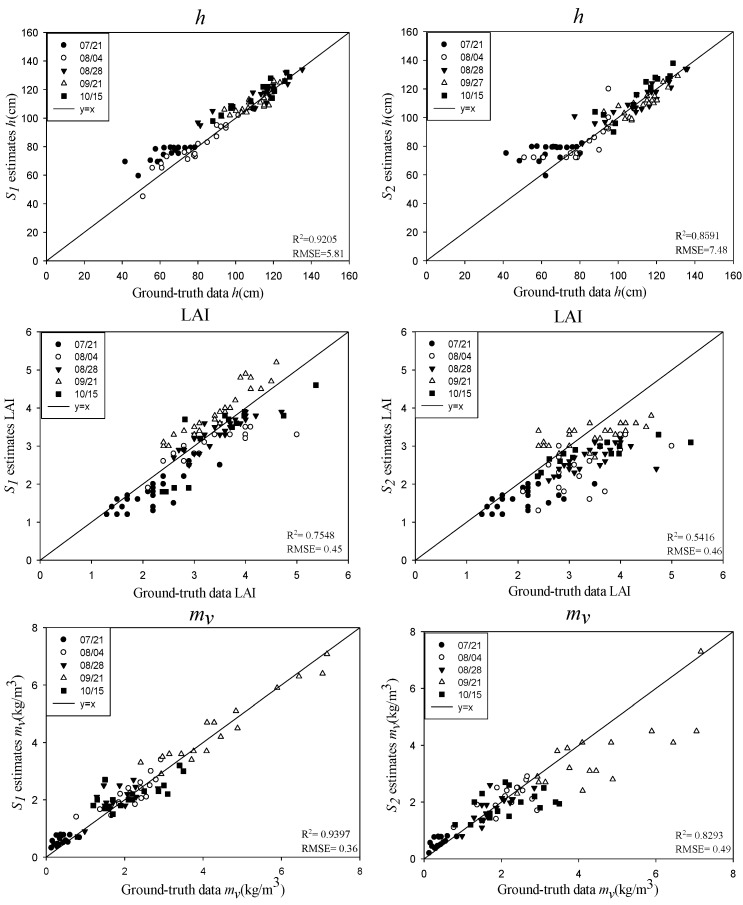

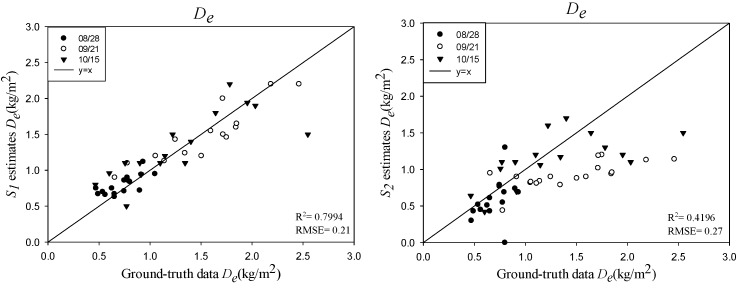
Results for the rice biophysical parameters using the Stokes parameters for the different phenological stages of rice.

**Figure 9:**
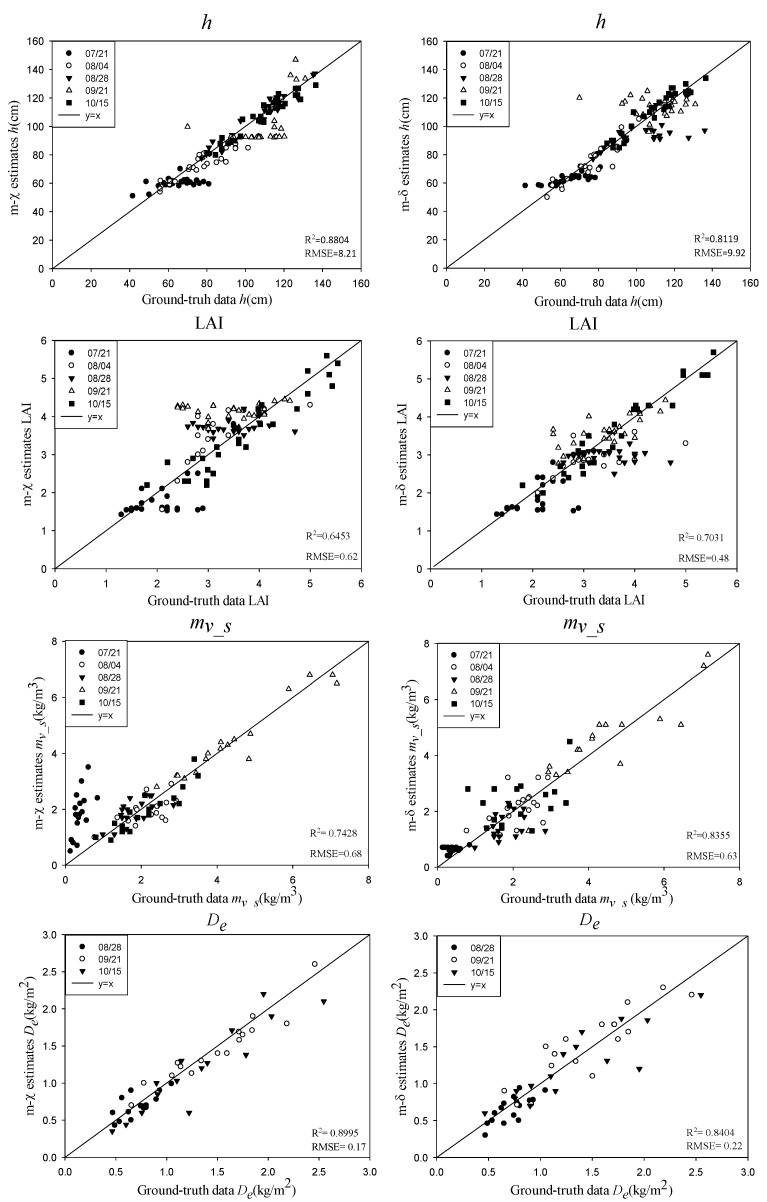
Results for the rice biophysical parameters using *m-χ* and *m-δ* decomposition for the different phenological stages of rice.

**Figure 10:**
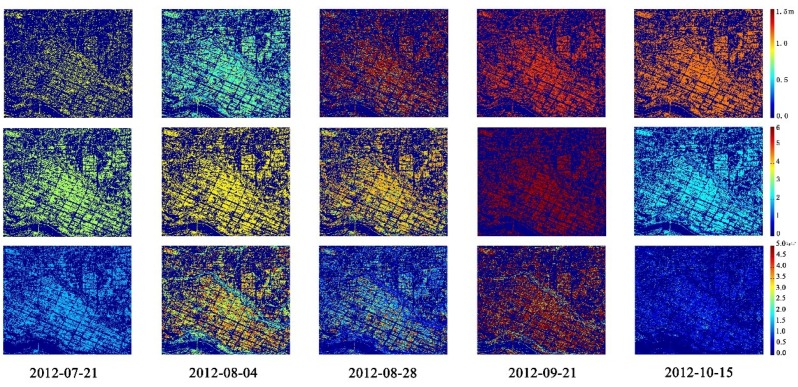
The inversion results for the rice biophysical parameters at five different phenological stages using the *m-χ* decomposition parameters. (The five images in the first row are the inversion results for *h*, the five images in the second row are the results for the leaf area index (LAI), and the five images in the third row are the results for *m_v_s_*).

**Figure 11:**
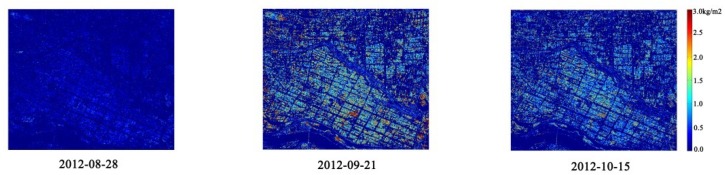
The inversion results for the rice biophysical parameter *D_e_* at three phenological stages using the *m-χ* decomposition parameters.

Based on the schemes of Section 3, the rice biophysical parameters, including the rice height (*h*), the LAI, the VWC of the rice canopy or the stem layer (*m_v_/m_v_s_*), and the biomass of the rice ear (*D_e_*), were estimated using the simulated CP SAR data. A total of 10 rice fields were used as training data for the GA, and the remaining twenty rice fields were used for validation. The estimated results of the rice biophysical parameters in different phenological stages were shown in Figure 7, Figure 8, Figure 9, Figure 10 and Figure 11.

### 4.1. Inversion of Rice Biophysical Parameters Using CP Backscattering Coefficients

Figure 7 shows the estimated results of the rice biophysical parameters using CP backscattering coefficients and the validation with the ground truth data. As the WCM treats the canopy as a water cloud, consisting of a collection of identical water particles, characterized by a uniform scattering phase function, the VWC of the rice canopy (*m_v_*) is identical to that of the stem layer (*m_v_s_*). In general, the inversion results of *h* and *m_v_* were superior to those of *D_e_* and the LAI, when the four CP backscattering coefficients (RH/RV/RR/RL) were used. The coefficient of determinations (*R^2^*) for *h* and *m_v_* were both above 0.81, and the root-mean-square errors (RMSEs) were less than 10 cm and 0.39 kg/m^3^, respectively. This indicated that the backscattering coefficients of the four compact polarizations were more sensitive to the rice height and the VWC of the rice canopy compared with the LAI and *D_e_*. Among the four compact polarizations (RH/RV/RR/RL), RV had the best performance in the inversion of *h* and *m_v_*. The preferable results with the RV polarization were mainly due to its sensitivity to the volume scattering of the rice canopy. For the inversion of *D_e_*, RH and RR got the accuracies of *R^2^* above 0.85 and a RMSE of less than 0.19 kg/m^3^, a little higher than that of RV but much better than that of RL. This suggested that RL was not as sensitive to the ear biomass as other compact polarizations. The reason is that the volume scattering was dominant in the ear layer, while RL was more related to the surface scattering. Compared with *h*, *m_v_*, and *D_e_*, the LAI had the poorest inversion results when using the backscattering coefficients of the four compact polarizations, of which RH was the best for the retrieval of the LAI, with a *R^2^* of 0.79 and an RMSE of 0.33.

### 4.2. Inversion of Rice Biophysical Parameters Using Stokes Parameters

Figure 8 shows the estimated results of the rice biophysical parameters using the Stokes parameters (*S*_1_ and *S*_2_) and the validation with the ground truth data. In general, similar to CP backscattering coefficients, the inversion results of *h* and *m_v_* were superior to those of *D_e_* and the LAI, when the Stokes parameters (*S*_1_ and *S*_2_) were used. The *R^2^* for *h* and *m_v_* were both above 0.85, and the RMSEs were less than 8 cm and 0.49 kg/m^3^, respectively. This indicated that the Stokes parameters (*S*_1_ and *S*_2_) were more sensitive to the rice height and the VWC of the rice canopy compared with the LAI and *D_e_*. In addition, based on the equation of the water cloud model and the idea of establishing the water cloud model, *h* and *m_v_* contributed a lot to the model, which also led to the high precision of the inversion of *h* and *m_v_*. Among the Stokes parameters (*S*_1_ and *S*_2_), *S*_1_ had the best performance in the inversion of *h* and *m_v_*. As can be seen from the above Equations (14) and (15) and Figure 4, *S*_1_ contained multi-polarization characteristics, which contained more energy than *S*_2_. The preferable results with *S*_1_ were mainly due to its sensitivity to the total scattering of the rice canopy. For the inversion of *D_e_*, *S*_1_ got the accuracies of *R^2^* of 0.79 and RMSE of 0.21 kg/m^3^, much better than that of *S*_2_ with a *R*^2^ of 0.41 and a RMSE of 0.27. The main scattering energy of the rice ear came from its volume scattering, indicating that *S*_2_ was not as good as *S*_1_ in the characterization of volume scattering characteristics. Especially on 21 September and 15 October, for the inversion of *D_e_*, the points of inversion results deviated from the line “y = x” significantly, which meant that the accuracy of *S*_2_ was far less than that of *S*_1_ in the dough stage and the mature stage. Compared with *h*, *m_v_*, and *D_e_*, the LAI had the poorest inversion results when using the Stokes parameters (*S*_1_ and *S*_2_), of which *S*_1_ was the best for the retrieval of the LAI, with a *R^2^* of 0.75 and a RMSE of 0.45.

### 4.3. Inversion of Rice Biophysical Parameters Using m-χ and m-δ Decomposition Parameters

Figure 9 shows the estimated results of the rice biophysical parameters using the *m-χ* and *m-δ* decomposition parameters and the validation with the ground truth data. In MWCM, the heterogeneity of the rice canopy and its phenological changes, as well as the double-bounce scattering between the rice canopy and the underlying surface were considered. Therefore, the VWC of the rice canopy (*m_v_*) is not identical to that of the stem layer (*m_v_s_*). In general, the inversion results of *h* and *D_e_* were superior to those of *m_v_s_* and the LAI, when the *m-χ* and *m-δ* decomposition parameters were used. The *R^2^* for *h* and *D_e_* were both above 0.81, and the RMSEs were less than 10 cm and 0.22 kg/m^2^, respectively. This indicated that the *m-χ* and *m-δ* decomposition parameters were more sensitive to the rice height and *D_e_* compared with the LAI and the VWC of the rice canopy. Moreover, there were close relations between *h* and *V_f_r_*, *V_f_s_*, *S_t_*, *S_g_r_*, *D_g_f_*, and *D_g_t_* in the MWCM. The three scattering types are all related to *h* and so make a significant contribution to the model, producing highly accurate results for the inversion of the rice height. Comparing the inversion results for *h* based on the two kinds of decomposition, the inversion accuracy based on the *m-χ* decomposition was higher than that based on the *m-δ* decomposition—the *R^2^* was 0.07 larger, and the RMSE was 1.7 cm smaller. 

According to Figure 6, the dominant scattering type for both the *m-χ* and *m-δ* decomposition is the volume scattering. Moreover, the scattering characteristics of the rice ear were mainly reflected in the volume scattering. Therefore, the *D_e_* inversion using the decomposition parameters had a higher accuracy, with an *R*^2^ above 0.8 and a RMSE below 0.22 kg/m^2^. Comparing the inversion results for *D_e_* based on the two kinds of decomposition, the inversion accuracy based on the *m-χ* decomposition was higher than that for the *m-δ* decomposition: the *R^2^* was 0.059 bigger, and the RMSE was 0.05 kg/m^2^ smaller. From 28 August to 15 October, *m-χ*-db was bigger than *m-δ*-db (see Figure 6) which is related to the effect of *D_g_e_* in the MWCM and indicates that *m-χ*-db is more sensitive than *m-δ*-db to *D_e_* inversion.

For the inversion of *m_v_s_*, the *m-δ* decomposition parameters got the accuracies of *R^2^* of about 0.83 and RMSE of 0.63 kg/m^3^, much better than that of the *m-χ* decomposition parameters. From Figure 9, it can be seen that both sets of inversion results produced overestimates on 21 July, with the overestimation by the *m-χ* decomposition being more obvious. This may be related to the three kinds of scattering components; also, at this stage, the double-bounce scattering and surface scattering values for *m-χ* were higher than those for the *m-χ* decomposition.

Compared with *h*, *m_v_s_*, and *D_e_*, the LAI had the poorest inversion results when using the *m-χ* and *m-δ* decomposition parameters. The *m-δ* decomposition parameters were better than the *m-χ* decomposition parameters for the retrieval of the LAI, with a *R^2^* of 0.7 and a RMSE of 0.48, but the difference in the accuracy was small: the *R^2^* was 0.05 higher, and the RMSE was 0.14 smaller. 

Figure 10 shows the inversion results for the rice biophysical parameters *h*, *m_v_s_*, and the LAI using the *m-χ* decomposition parameters. It can be seen from Figure 10 that the inversion results of *h*, *m_v_s_*, and the LAI have low values in the elongation stage (21 July), and the inversion results increase from the elongation stage (21 July) to the booting stage (4 August). The rice height was about 60–80 cm, the LAI is around 2–3, and the value of *m_v_s_* is about 0.5–3 in the elongation stage and the booting stage. During the heading stage (28 August) and the dough stage (21 September), the three biophysical parameters reached their peak values. The peak height of rice was then about 120 cm, the peak LAI is around 3–5, and the peak value of *m_v_s_* is about 3–4 kg/m^3^. These values then decreased slightly during the mature stage (15 October). This was because the rice as mature, the plant turned yellow, the stem contained less water, the size of the ear layer increased, and the rice got shorter. The inversion results for the other CP parameters show similar patterns to those seen in Figure 10.

Figure 11 shows the inversion results for *D_e_* using the *m-χ* decomposition parameters. Because the ears began to form during the heading stage (28 August), *D_e_* then is low. *D_e_* reached its peak during the dough stage (21 September). The rice ears on September 21 were nearly mature, and so, the value *D_e_* remained basically unchanged on 15 October. The results for inverting *D_e_* using the other CP parameters show similar patterns to those seen in Figure 11.

## 5. Conclusions

The objective of this study was to investigate the capability of CP SAR data in the inversion of rice biophysical parameters during the growing season. Five temporal RADARSAT-2 fully polarimetric SAR datasets were used to simulate CP SAR data, and the CP parameters, such as backscattering coefficients, Stokes vectors and decomposition parameters were extracted in each acquisition. Then, the inversion models of rice biophysical parameters with CP SAR data were developed by introducing a classic semi-empirical model, water cloud model, and a modified WCM in which the heterogeneity of the rice canopy and its phenological changes as well as the double-bounce scattering between the rice canopy and the underlying surface were considered. Finally, rice biophysical parameters, including rice height (*h*), LAI, the VWC of the rice canopy or the stem layer (*m_v/_m_v_s_*), and biomass of the ear (*D_e_*), were obtained, and the validation was conducted using the ground truth data. The detailed conclusions are as follows:

(1) Using the four CP backscattering coefficients (RH/RV/RR/RL), the inversion results of *h* and *m_v_s_* were superior to those of LAI and *D_e_*, with the *R*^2^ above 0.81 and the RMSE less than 10 cm and 0.39 kg/m^3^, respectively. RV had the best performance in the inversion of *h* and *m_v_s_*. For *D_e_*, RH and RR performed better than RV and RL, giving an *R*^2^ above 0.85 and an RMSE of less than 0.18 kg/m^2^. Again, the inversion results for the LAI were the poorest, with RV and RL giving better results than RH and RR. For RL, the *R*^2^ was 0.73, and the RMSE was less than 0.55.
(2) Using the Stokes parameters, the inversion results for the four rice biophysical parameters were good. For *h*, the *R*^2^ had a value of about 0.92, and the RMSE was less than 5.8 cm. For *m_v_s_*, the *R*^2^ was about 0.93, and the RMSE was less than 0.36 kg/m^3^. For *D_e_*, the *R*^2^ was about 0.80, and the RMSE was less than 0.21 kg/m^2^. The inversion results for the LAI were the poorest, with an *R*^2^ of 0.75 and an RMSE of up to 0.45.
(3) Using the *m-χ* and *m-δ* decomposition parameters, the inversion accuracy for *h* and *D_e_* was higher than for *m_v_s_* and the LAI. For *h*, the coefficient of determination, *R*^2^, was about 0.88, and the RMSE was less than 8.2 cm. For *D_e_*, the *R*^2^ was 0.89, and the RMSE was less than 0.17 kg/m^2^. For *m_v_s_*, the coefficient of determination was 0.83, and the RMSE was less than 0.6 kg/m^3^. As before, the inversion results for the LAI had the lowest accuracy, with an *R*^2^ of 0.73 and an RMSE up to 0.55. Overall, the *h* and *D_e_* inversion results obtained using the *m-χ* decomposition parameters were better than those obtained using the *m-δ* decomposition. For LAI and *m_v_s_*, the accuracy obtained using the *m-δ* decomposition was slightly higher than that found using the *m-χ* decomposition. In the early stages of rice growth, *m_v_s_* was slightly overestimated; in the case of the *m-χ* decomposition parameters, this overestimation was even greater.
(4) In general, *S*_1_, RV, RL, and the *m-χ* decomposition parameters proved to be more suitable for the inversion of *h*, and high inversion accuracy was obtained. *S*_1_, RH, RV, RR, and RL are more suitable for inverting *m_v_*. RH, RR, and the decomposition parameters are more suitable for inverting *D_e_*.

In this paper, the capability of CP SAR data in the inversion of rice biophysical parameters has been proven, especially for the inversion of *h*, *D_e_*, and *m_v_s_*/*m_v_*, with an *R*^2^ of higher than 0.89 and an RMSE of less than 10 cm, 0.68 kg/m^3^, and 0.27 kg/m^2^. Further validation and improvement will be conducted with the subsequent dataset of this site and the dataset of other regions.

## Figures and Tables

**Table 1:** Details of the full polarimetric RADARSAT-2 SAR data.

Date	Mode	Product	Incidence Angle (°)	Pixel Spacing (A × R, m)	Phenology Stage
21 July 2012	FQ20W ^1^	SLC ^2^	38–41	5.2 × 7.6	Elongation
4 August 2012	FQ9W	SLC	27–30	5.2 × 7.6	Booting
28 August 2012	FQ9W	SLC	27–30	5.2 × 7.6	Heading
21 September 2012	FQ9W	SLC	27–30	5.2 × 7.6	Dough
15 October 2012	FQ9W	SLC	27–30	5.2 × 7.6	Mature

^1^ FQW = fine quad-polarimetry wide, 20 or 9 is the number of the beam position, which is related to the incidence angles; ^2^ SLC = single look complex [21].

**Table 2:** Rice scattering mechanisms for different phenological stages.

Phenological Stages	Underlying Surface	Rice Part Scattering					Space Part Scattering				
Seedling	Water surface	*S_g_r_*	*V_f_r_*				*S_g_s_*	*D_g_f_*			
Tillering to Booting	Water surface	*S_g_r_*	*V_f_r_*	*S_t_*	*D_g_t_*		*S_g_s_*	*D_g_f_*	*V_f_s_*		
Heading to Flowering	Moist soil	*S_g_r_*	*V_f_r_*	*S_t_*	*D_g_t_*	*V_e_r_*	*S_g_s_*		*V_f_s_*	*D_g_e_*	
Dough to Mature	Moist soil	*S_g_r_*	*V_f_r_*	*S_t_*	*D_g_t_*	*V_e_r_*	*S_g_s_*		*V_f_s_*	*D_g_e_*	*V_e_s_*
